# Draft Genome Sequence of the Cellulolytic Bacterium Clavibacter sp. CF11, a Strain Producing Cold-Active Cellulase

**DOI:** 10.1128/genomeA.01304-14

**Published:** 2015-01-02

**Authors:** Ying Du, Bo Yuan, Yonghui Zeng, Jianyu Meng, Heng Li, Ruigang Wang, Guojing Li, Fuying Feng

**Affiliations:** a Institute for Applied & Environmental Microbiology, Life Sciences College, Inner Mongolia Agriculture University, Huhhot, China; b Life Sciences and Technology College of Inner Mongolia Normal University, Huhhot, China; c Institute of Microbiology CAS, Opatovický Mlýn, Třeboň, Czech Republic

## Abstract

Clavibacter sp. strain CF11, which was isolated from soil at a tomato-planting greenhouse in Inner Mongolia, North China, has a high capability for producing cold-active cellulase at low temperatures. Here, we report the draft genome sequence of strain CF11, which comprises 2,437 protein-coding sequences and 49 RNA-coding sequences.

## GENOME ANNOUNCEMENT

Cellulose is one of the most promising alternatives to fossil fuels and is one of the most abundant and renewable biopolymers on the earth. Cellulose degradation under low temperature accounts for a large proportion of the carbon cycle in nature (1), while cold-active cellulase may contribute to energy saving and therefore is very attractive in some application fields at low temperatures (2). We isolated a cellulolytic and low-temperature-tolerant bacterium, strain CF11, belonging to Clavibacter, on 16S rRNA and *gyrB* genotyping, from soil at a tomato-planting greenhouse in Inner Mongolia, North China. The 16S rRNA gene sequence of CF11 shows 98.3% identity to that of the Clavibacter michiganensis strain LMG 3663. The strain shows a high carboxymethylcellulose (CMC) enzyme activity (56 µmol·ml^-1^·min^-1^ at 10°C; optimal, 60 µmol·ml^-1^·min^-1^ at 22°C), which is different from the optimal activity of about 37°C seen in other Clavibacter spp. (3), and it has potential application at low temperatures. The genome sequencing may be helpful in understanding and developing the cold-active cellulase of CF11.

We extracted DNA using a genomic DNA purification kit (Tiangen Biotech Co., Beijing, China), constructed a 300-bp DNA library for Illumina sequencing technology, and employed an Illumina HiSeq 2000 platform for sequencing. Approximately 11.2 Gbp of raw data of 101-bp-long paired-end reads were generated. A total of 6,140,800 × 2 reads were subjected to quality control and trimming on the Galaxy server (4) and then were *de novo* assembled into contigs using the Velvet program (version 1.2.08) (5). Various hash lengths between 31 and 91 were tested, and an optimal assembly was achieved with a *k*-mer size of 27. Contigs <300 bp long were removed from the final assembly. The genome coverage was 365.3×. Annotation was performed with the RAST (6) and BASys (7) servers.

The draft genome comprises 45 contigs, with an *N*_50_ value of 165,058 bp and an *N*_90_ contig length of 45,901 bp. The total length is 2.97 Mb, containing 2,437 protein-coding sequences (CDS) and 49 RNA-coding sequences detected after manual inspection of the annotations using the RAST server (6). Among these, 45 tRNA sequences and 4 rRNA clusters were found. The G+C content is 73.56%. Cellulose-degrading enzymes were annotated: two galactosidases, two endo-1,4-β-xylanases, two β-glucosidases, and two endo-1,4-β-glucanases. These cellulolytic and hemicellulolytic enzymes have biotechnological potentials in various industries.

### Nucleotide sequence accession numbers.

The genome sequence of C. michiganensis CF11 has been deposited in GenBank under the accession no. JROD00000000. The version described in this paper is the first version, JROD01000000.

## References

[1] Kasana RC, Gulati A 2011 Cellulases from psychrophilic microorganisms: a review. J Basic Microbiol 51:572–579. doi:10.1002/jobm.201000385. 21656807

[2] Bhat A, Riyaz-Ul-Hassan S, Ahmad N, Srivastava N, Johri S 2013 Isolation of cold-active, acidic endocellulase from Ladakh soil by functional metagenomics. Extremophiles 17:229–239. doi:10.1007/s00792-012-0510-8. 23354361

[3] Baer D, Gudmestad NC 1995 *In vitro* cellulolytic activity of the plant pathogen *Clavibacter michiganensis* subsp. sepedonicus. Can J Microbiol 41:877–888. doi:10.1139/m95-121. 8590403

[4] Goecks J, Nekrutenko A, Taylor J, Galaxy T 2010 Galaxy: a comprehensive approach for supporting accessible, reproducible, and transparent computational research in the life sciences. Genome Biol 11:R86. doi:10.1186/gb-2010-11-8-r86. 20738864 PMC2945788

[5] Zerbino DR, Birney E 2008 Velvet: algorithms for *de novo* short read assembly using de Bruijn graphs. Genome Res 18:821–829. doi:10.1101/gr.074492.107. 18349386 PMC2336801

[6] Aziz RK, Bartels D, Best AA, DeJongh M, Disz T, Edwards RA, Formsma K, Gerdes S, Glass EM, Kubal M, Meyer F, Olsen GJ, Olson R, Osterman AL, Overbeek RA, McNeil LK, Paarmann D, Paczian T, Parrello B, Pusch GD, Reich C, Stevens R, Vassieva O, Vonstein V, Wilke A, Zagnitko O 2008 The RAST server: Rapid Annotations using Subsystems Technology. BMC Genomics 9:75. doi:10.1186/1471-2164-9-75. 18261238 PMC2265698

[7] Van Domselaar GH, Stothard P, Shrivastava S, Cruz JA, Guo A, Dong X, Lu P, Szafron D, Greiner R, Wishart DS 2005 BASys: a Web server for automated bacterial genome annotation. Nucleic Acids Res 33:W455–W459. doi:10.1093/nar/gki593. 15980511 PMC1160269

